# Prevalence of G6PD Deficiency in Neonatal Sepsis in Iran

**Published:** 2013-09-15

**Authors:** Soheila Zareifar, Narjes Pishva, Mohamadreza Farahmandfar, Shahab Benaei, Nader Cohan

**Affiliations:** 1 Hematology Research Center; 2 Neonatology Research Center, Shiraz University of Medical Sciences, Shiraz, Iran

**Keywords:** G6PD Deficiency, Neonate, Sepsis, Prevalence

**Dear editor,**

In Iran, according to WHO, the prevalence of G6PD enzyme deficiency is 10-14.9%^[^^1^^]^. An epidemiological study showed in the Fars province (southern Iran) 12% of males and 1.8% of females are G6PD deficient^[^^2^^]^. Initial neutrophil bactericidal activity depends on oxygen free radical production by the NADPH oxidase. In G6PD deficient state, decreasing the production of neutrophil NADPH has been reported. Few studies so far on the G6PD deficiency and increased chance of infection was carried out^[^^3^^]^. Regarding the higher probability of septicemia in neonates with G6PD deficiency, several studies were done^[^^4^^,^^5^^]^. Some studies showed it may be due to lower level of the G6PD enzyme activity in white blood cells^[^^3^^]^. In contrast other studies revealed that G6PD activity is higher in premature infants with gestational age of 29-32 weeks than in term neonates and this does not interfere with diagnosis of G6PD deficiency.

 In a prospective study from April 2007 till April 2009, in teaching hospitals of Shiraz University of Medical Sciences 150996 neonates were screened for G6PD enzyme activity in the first week of life. From these, 660 neonates have been admitted and screened for sepsis based on the clinical features in neonatal intensive care units. Parental written informed consent form was obtained from all patients for inclusion in the study. The Shiraz University of Medical Sciences Ethics Committee approved the study. 

 Neonatal sepsis was defined as a clinical syndrome characterized by signs and symptoms of infection with or without accompanying bacteremia in the first month of life. Patients were divided into three groups:

1) Non-sepsis cases based on clinical symptoms and paraclinical data of following days were excluded.

2) Suspected sepsis was diagnosed according to clinical symptoms and laboratory abnormalities including 5000≤WBC≥25000, C- reactive protein (CRP) >6 mg/lit, immature neutrophils/total neutrophils >0.2, and negative blood cultures.

3) Definite sepsis diagnosis was established with positive blood cultures. 

 We enrolled second and third groups in the study. 

 For all neonates in whom sepsis was suspected, screening tests including complete blood count, CRP, and micro erythrocyte sedimentation rate (ESR), blood culture, urine culture, cerebrospinal fluid (CSF) analysis and culture were performed. G6PD activity was measured by qualitative method, fluorescent spot test, using kits (Kymia Pajohan, Iran) with 99% specificity and sensitivity.

 The prevalence of G6PD deficiency in male and female were 10932 (14.09%) and 8650 (11.77%)

**Table 1:** The prevalence of G6PD deficiency and sepsis among 150996 newborns in southern Iran

	**Total**	**Male**	**Female**
**Newborns**	150996	77536 (51.34%)	73460 (48.66%)
**G6PD deficiency**	19582 (12.9%)	10932 (14.09%)	8650 (11.77%)
**Sepsis**	110 (0.07%)	68 (0.04%)	42 (0.02%)
**G6PD deficiency among ** **newborns with sepsis**	15 (13.6%)	10 (9%)	5 (4.5%)

respectively. The overall incidence of G6PD enzyme deficiency was 12.96%. Of the total neonates 660 neonates suspected to have sepsis were referred to neonatal intensive care units, but finally after exclusion of other causes such as metabolic disorders and hypoxic ischemic encephalopathy, 110 patients (68 males and 42 females) were admitted and screened with impression of sepsis. The G6PD enzyme deficiency was proved in 15 (13.6%) of them. There was no statistically significant difference between male and female gender in this group (*P*=0.78). Table 1 shows the pre-valence of G6PD deficiency and sepsis among 150996 newborns in southern Iran. Few studies on the G6PD deficiency and increased chance of infection were so far carried out. Several reasons for this relationship are considered. 

 Our study showed that G6PD deficiency does not increase the chance of neonatal septicemia. Because of different variety of G6PD deficiency in different populations more studies by larger study groups is recommended for evaluation of relationship between G6PD deficiency and bacterial infection and sepsis.
